# Fifteen–year record of soil temperature at the Bear Brook Watershed in Maine

**DOI:** 10.1038/sdata.2018.153

**Published:** 2018-07-31

**Authors:** Kaizad F. Patel, Sarah J. Nelson, Cheryl J. Spencer, Ivan J. Fernandez

**Affiliations:** 1 School of Forest Resources, University of Maine, 5755 Nutting Hall, Orono, ME 04469, USA; 2 Climate Change Institute, University of Maine, 5764 Sawyer Research Center, Orono, ME 04469, USA

**Keywords:** Environmental sciences, Climate sciences, Forest ecology

## Abstract

This paper describes a record of air and soil temperature collected from 2001 to 2016 in temperate forests at the Bear Brook Watershed in Maine (BBWM). BBWM is a long-term research site established to study the response of forest ecosystem function to various environmental disturbances, including chronic acidic deposition. Replicate HOBO data loggers were deployed in BBWM’s two forest types (coniferous and deciduous), to record temperatures at four positions: (1) air temperature, 100 cm above the forest floor; (2) surface organic soil, 2 cm below the forest floor surface; (3) mineral soil, 10 cm below the organic–mineral horizon interface; and (4) mineral soil, 25 cm below the organic–mineral horizon interface. Data were recorded every three hours, and these raw data were used to compute daily maximum, daily minimum, daily average, and monthly average values. This fifteen–year record represents one of the few readily–available soil temperature datasets in the region, and provides information on long-term changes in climatology, and seasonal and episodic weather patterns.

## Background & Summary

Soil temperature is an important driver of terrestrial biogeochemical processes. Soil temperature influences microbial and plant activity^1–4^, and therefore plays a critical role in the cycling of nutrients like carbon and nitrogen^5–11^. Phenological changes occurring during seasonal transitions are often strongly influenced by changing soil temperatures^12–14^. Despite the importance of soil temperature for ecosystem function, long-term datasets of soil temperature are not commonly available, even fewer are available at multiple soil depths, and models often use air temperature as a proxy or basis for simulations of soil temperature^15–19^. While air and soil temperatures are often well correlated, soil temperature is also influenced by environmental variables such as forest composition and canopy cover^20,21^, snow cover^22–24^, and soil moisture^15,25^, which may not be adequately parameterized into the models to provide suitable pedotransfer functions. Additionally, disturbances can alter soil temperatures on short temporal scales^20,23^ due to changes in canopy structure and understory vegetation, organic debris on the forest floor, or snowpack loss in winter, and these may not be reflected in the soil temperature simulations. Access to long-term datasets of empirical soil temperature measurements is therefore valuable when studying ecosystem processes over short and long time intervals, made even more important in a time of accelerating changes in the climate including warming temperatures, the intensification of the hydrologic cycle, and increased inter- and intra-annual variability in weather^26–28^.

**Figure 1:**
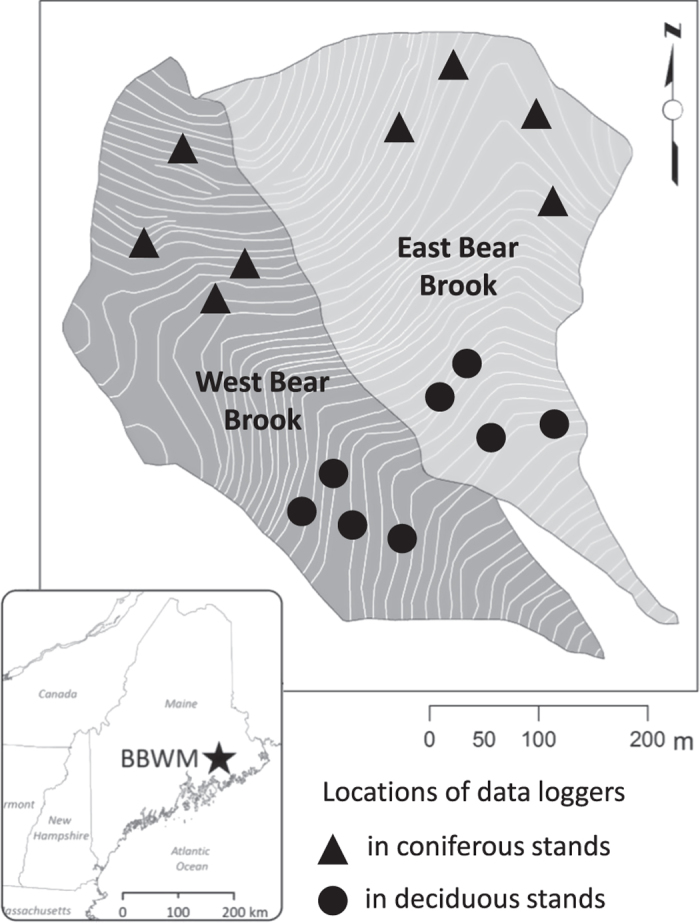
Location and layout of the Bear Brook Watershed in Maine (BBWM), with paired watersheds East Bear Brook (light gray) and West Bear Brook (dark gray). The markers represent locations of the HOBO temperature data loggers described in this paper, with circles representing data loggers in deciduous stands, and triangles representing data loggers in the coniferous stands. Contour lines represent 20-foot intervals.

The objective of this paper is to provide a 15-year dataset of soil temperature from the Bear Brook Watershed in Maine (BBWM). BBWM is a long-term whole-watershed acidification experiment in eastern Maine, USA (44°52'N, 68°06'W), established to study the effects of elevated nitrogen and sulfur deposition on ecosystem processes (Fig. 1). BBWM is comprised of paired watersheds, the reference East Bear Brook (EB, 11.0 ha) and the manipulated West Bear Brook (WB, 10.3 ha) that received bimonthly ammonium sulfate additions from 1989 to 2016 (ref. 29). Vegetation is similar in both watersheds, with lower elevations dominated by deciduous species including *Fagus grandifolia* (American beech), *Acer saccharum* (sugar maple), and *Acer rubrum* (red maple), and higher elevations dominated by coniferous species including *Picea rubens* (red spruce) and *Abies balsamea* (balsam fir). Thus, each watershed is split into two compartments, with a total of four compartments at the site (East Bear–deciduous, East Bear–coniferous, West Bear–deciduous, and West Bear–coniferous). Soils are coarse-loamy, mixed, frigid Typic and Aquic Haplorthods (Lyman, Tunbridge, Rawsonville, Dixfield, Colonel series)^30,31^. Since 2001, air and soil temperatures have been recorded at the site to gain a better understanding of the biogeochemical processes occurring in the watersheds. Temperature has been measured in the organic and underlying mineral soil horizons to characterize temporal variability in soil temperature with depth^32^. Soil temperatures have also been measured in both forest types to account for differences in canopy cover. In this paper, we describe the instrumentation, data collection, and data handling for this temperature dataset.

## Methods

### Instrumentation

Temperature was recorded using Onset HOBO data loggers H8 and U12, with TMC1-HD and TMC6-HD temperature sensors (Onset Computer Corporation, Bourne, MA, USA). In July 2001, four data loggers were deployed in each forest type at the site (two data loggers in each compartment). From June 2003 to August 2007, four additional data loggers were deployed in each forest type to examine spatial heterogeneity in temperature measurements (total n=8 per forest type). Due to limited availability of resources, after August 2007, replication was reduced to four data loggers in each forest type. We tested for the effect of replication size using linear mixed effect models, and replication size did not significantly alter the final means. Further details are included in the Technical Validation section, and results are reported in Table 1.

Each data logger was equipped with four sensors to measure temperature at four positions: (1) air temperature, 100 cm above the forest floor surface; (2) surface organic (O) horizon, where the sensor was placed 2-3 cm below the forest floor surface; (3) 10 cm below the interface of organic and mineral horizons, which corresponded to placement in the B horizon; and (4) 25 cm below the interface of organic and mineral horizons, which corresponded to the lower B or BC horizon. Data loggers were mounted on wooden stakes and enclosed in PVC towers for protection from damage by wildlife. Air and soil temperatures were recorded year-round, every three hours, beginning at 12:00 AM. The data loggers were inspected at the site every four to six months, and batteries and desiccant were replaced as needed. Additional information on data logger setup and experimental design can be found in Fernandez *et al.*^33^

### Data analysis and processing

#### Removal of outliers

We used methods described in the literature to test for variance in our data, and to detect outliers^34,35^. We established an acceptable temperature range of −50 to+50 °C, since historical air temperature data from National Oceanic and Atmospheric Administration (NOAA) weather stations at multiple locations in Maine (Acadia National Park, GHCND:USC00170100; Bangor, GHCND:USW00014606; Caribou GHCND:USW00014607)^36,37^ were always within this range. The data flagged by this process were an order of magnitude greater than our acceptable limits (+/- 500 to 900 °C), and we excluded these data points as spurious.

We calculated standard deviation (SD) on the long-term raw data to examine the variation of the data and detect statistical outliers. Values that exceeded the range of *mean*±*3 SD* were flagged as potential outliers, and were then inspected manually. When these outliers were consistent across multiple sensors, we interpreted them as “real values”, because they represented days that were unusually cold or warm compared to the long-term average. If the outliers were restricted to only one sensor, they were excluded.

#### Internal consistency check

We performed internal consistency checks on air temperature, to test that maximum>mean>minimum. Maximum and minimum values were equal for some sensors during winter months, indicating that those sensors were buried under snow. We excluded those values, since they did not represent air temperatures. We did not perform a similar check for soil temperatures, because soil temperatures often show little to no fluctuation (for instance, under snowpack).

#### Data processing

We calculated daily maximum, minimum, and average values for each replicate sensor. We performed correlation analysis on all replicates within each forest type to check for spatial consistency. This was done for the period 2003–2007, since all replicate loggers were active during this period. All replicates were well correlated (r=1.0, p<0.01). We averaged values across all replicates to compute daily maximum, daily minimum, and daily mean temperature for each forest type. Daily average values were used to compute monthly average values.

#### Missing values

The dataset contains some missing values, most notably for five months in 2012. This was a result of equipment malfunctions coupled with logistical issues that prevented maintenance of the data loggers during this period. Missing data are indicated by blank entries. We have left these gaps unfilled, and have not used climate models to estimate the missing data, because our objective is to provide a dataset of recorded temperatures.

## Data Records

Daily and monthly data are available online (Data Citation 1), in ten tab-delimited text files. Each file name begins with “Bear_Brook_Watershed_” and is followed by a suffix describing the nature of the data, i.e. air or soil; organic soil, mineral soil at 10 cm depth or 25 cm depth; and deciduous or coniferous forest (Table 2).

**Figure 2:**
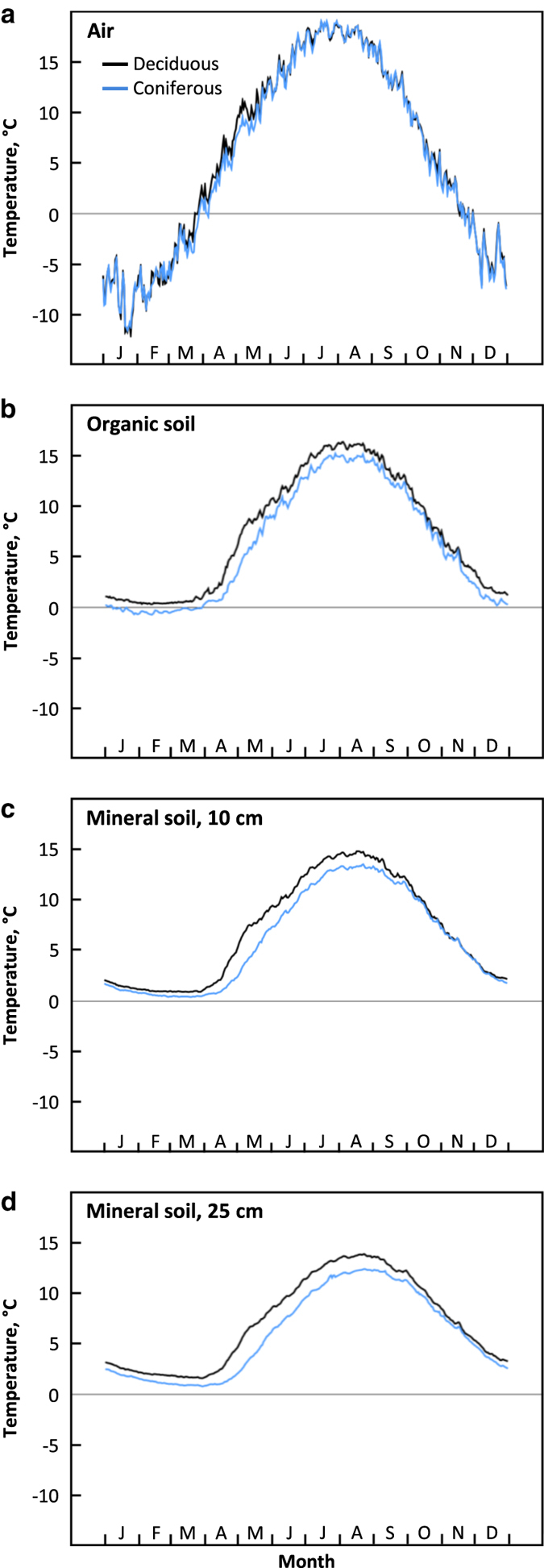
Daily values of temperature averaged across 16 years (2001–2016) for air, organic soil, and mineral soil at 10 and 25 cm depths.

A summary of the 16-year record is presented in Table 3 and Fig. 2, and these highlight the effect of vegetation and the vertical stratification of temperature. Deciduous stands had higher soil temperatures than coniferous stands, most prominent during spring and summer. This is likely due to a shading effect under the dense coniferous canopy. Air temperatures showed greatest variability and temperature ranges, while deep mineral soils showed the least variability.

## Technical Validation

### Quality assurance procedures on data loggers

**Figure 3:**
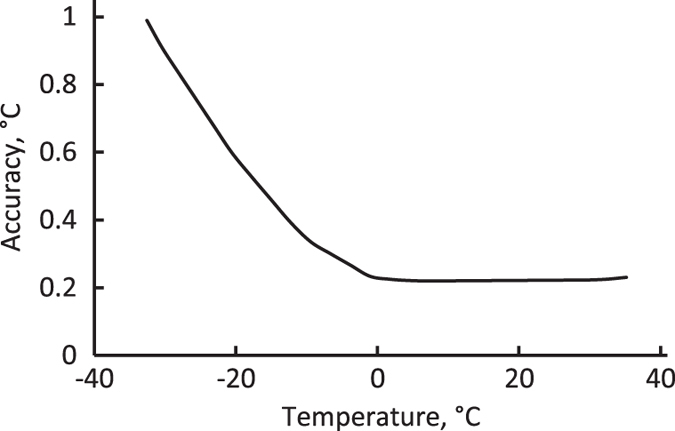
Plot of accuracy vs. measured temperature for TMCx Soil Temperature Sensors, as provided by Onset Computer Corporation.

The data loggers and sensors were calibrated by Onset Computer Corporation, and were accurate to±0.2 °C above 0 °C, and accuracy declined from±0.2 °C to±0.9 °C between 0 °C and -30 °C (Fig. 3). Additionally, we tested all data loggers and sensors for accuracy prior to deployment, by immersing the sensors in an ice bath, as described at http://www.onsetcomp.com/support/tech-notes/quick-temp-accuracy-check-ice-bath. This method operates on the principle that a mixture of ice and water maintains its temperature at ~0.01 °C, the triple point of water. All sensors recorded the temperature of the ice bath as 0.00±0.01 °C, and were therefore determined to be acceptable for deployment in the field.

### Quality control procedures on temperature data

We analyzed the processed data (daily maximum, minimum, average) using statistical methods described in the literature^34,35,38,39^.

#### Spatial consistency among sensors

We conducted paired correlations on processed data among data loggers. All replicates within each forest type were strongly correlated (r=1.00, p<0.01) suggesting consistency among replicates.

#### Testing for bias and the effect of replication

To determine if the degree of replication influenced our values, we compared daily mean temperatures obtained using varying replication sizes. Eight replicate sensors were active during the period 2001–2003, and we randomly subsampled from these sensors to get replication sizes from four to eight. We analyzed these data using linear mixed effects models (fixed effect=replication level; random effect=forest; correlation=AR1 to account for autocorrelation; n=3000). The null hypothesis (that there was no significant effect of replication size) was proven correct. Statistical results as well as least-square means are provided in Table 1. To test if the mean was significantly biased by any single sensor, we calculated the mean using all eight sensors, and compared it with the mean of seven sensors, calculated iteratively by excluding one sensor at a time. All combinations were statistically similar, and no single sensor was found to significantly influence the overall means. These tests were run on data recorded during the period June 2003–August 2007. Detailed results can be found in Table 4.

#### Consistency with NOAA station data

**Figure 4:**
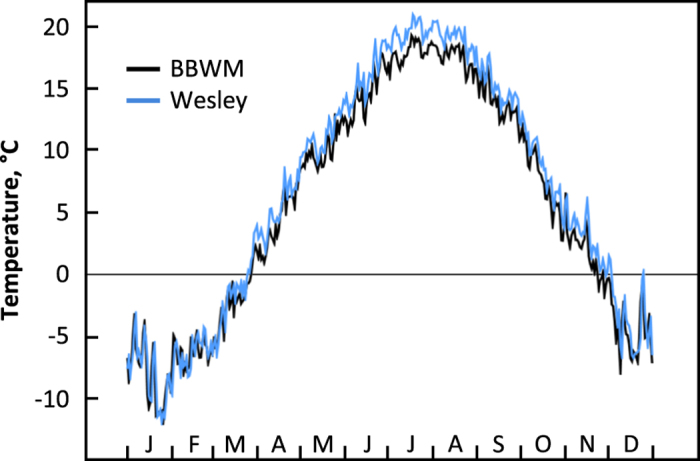
Daily mean air temperatures for BBWM and the Wesley weather station (NOAA station GHCND:USC00179294). Data are averaged across the 16 years of our study, 2001–2016.

We compared daily maximum and minimum air temperatures with records from the NOAA station at Wesley, ME (44.95 °N, 67.67 °W, GHCND:USC00179294)^36,37^, which is 35.41 km from our research site. The data from the two sites were well correlated (r=0.94, p<0.01), suggesting that the air temperature dataset for BBWM was consistent with the nearest weather station temperature record in the region (Fig. 4). Our recorded air temperature was statistically lower than Wesley values during the growing season and fall, which we attribute to canopy shading.

## Usage Notes

We expect that this dataset would be useful to researchers and professionals who need access to long-term temperature datasets to examine intra- or inter-annual trends in the region. Additionally, our data could be used to parameterize and/or validate climate models that predict soil temperature and soil function.

The goal of this work was to obtain a continuous air and soil temperature dataset over 16 years. However, there are limited periods without data, and users should be careful to note those periods in their work. Additionally, it should be noted that this dataset does not represent all possible site conditions for the entire watershed. The measurement locations accurately represent the moderate to well-drained forest soils that dominate the landscape of these watersheds, but sensors were not deployed in spatially minor but divergent site conditions such as in the relatively narrow riparian zone along streams, shallow to bedrock soils in the upper reaches of the watershed, or minor soils along the ridgeline of the watershed divide.

## Additional information

**How to cite this article**: Patel, K. F. *et al*. Fifteen–year record of soil temperature at the Bear Brook Watershed in Maine. *Sci. Data* 5:180153 doi: 10.1038/sdata.2018.153 (2018).

**Publisher’s note**: Springer Nature remains neutral with regard to jurisdictional claims in published maps and institutional affiliations.

## Supplementary Material



## Figures and Tables

**Table 1:** Least square mean temperatures and results from linear mixed-effects models testing the effect of replication size.

**Replication**	**Least square mean temperature (°C)**			
	**Air**	**Organic soil**	**Mineral soil, 10 cm**	**Mineral soil, 25 cm**
4	5.79±0.19	6.67±0.11	6.49±0.10	6.41±0.08
5	5.77±0.19	6.65±0.11	6.49±0.10	6.40±0.08
6	5.75±0.19	6.64±0.11	6.54±0.10	6.43±0.08
7	5.51±0.19	6.90±0.11	6.21±0.11	6.33±0.08
8	5.81±0.19	6.67±0.11	6.50±0.10	6.40±0.11
φ (autocorrelation)	0.9863	0.9971	0.9983	0.9983
F-value from LME	0.0415	0.3717	0.0986	0.4893
p-value from LME	0.9967	0.8290	0.9829	0.7437

**Table 2:** Summary of data files available.

**File ID suffix**	**Time scale**	**Forest type**	**Air or soil**	**Temperature statistic**
air_dec	Daily	Deciduous	Air	Maximum, minimum, average
soil_org_dec	Daily	Deciduous	Soil (organic)	Maximum, minimum, average
soil_10_dec	Daily	Deciduous	Soil (mineral, 10 cm)	Maximum, minimum, average
soil_25_dec	Daily	Deciduous	Soil (mineral, 25 cm)	Maximum, minimum, average
air_con	Daily	Coniferous	Air	Maximum, minimum, average
soil_org_con	Daily	Coniferous	Soil (organic)	Maximum, minimum, average
soil_10_con	Daily	Coniferous	Soil (mineral, 10 cm)	Maximum, minimum, average
soil_25_con	Daily	Coniferous	Soil (mineral, 25 cm)	Maximum, minimum, average
air_soil_mean_dec	Monthly	Deciduous	Air, soil (all depths)	Average
air_soil_mean_con	Monthly	Coniferous	Air, soil (all depths)	Average

**Table 3:** Summary of the data record over 16 years of monitoring.

	**Mean**	**SE**	**Maximum**	**Minimum**	**Range**
*Deciduous forest*					
Air	6.30*	0.03	38.30	−30.20	68.50
Organic soil	7.53*	0.02	22.50	−11.70	34.20
Mineral soil, 10 cm	7.30*	0.01	18.70	−2.44	21.14
Mineral soil, 25 cm	7.43*	0.01	19.40	−1.51	20.90
*Coniferous forest*					
Air	5.81	0.03	35.30	−36.50	71.70
Organic soil	6.55	0.02	23.60	−11.70	35.30
Mineral soil, 10 cm	6.18	0.01	17.50	−3.37	20.90
Mineral soil, 25 cm	5.93	0.01	16.80	−1.51	18.30
Asterisks denote significant differences between forest types at α=0.05.					

**Table 4:** Results from tests to check if the mean was biased by a single sensor.

	**Mean Temperature, °C**			
	**Air**	**Organic soil**	**Mineral soil, 10 cm**	**Mineral soil, 25 cm**
*Deciduous stands*				
all 8 sensors	6.12	7.18	7.15	7.14
exclude sensor D1	6.05	7.15	7.07	7.00
exclude sensor D2	6.06	7.13	7.05	7.10
exclude sensor D3	6.16	7.15	7.13	7.10
exclude sensor D4	6.09	7.14	7.12	7.10
exclude sensor D5	6.13	7.26	7.24	7.23
exclude sensor D6	6.14	7.18	7.13	7.17
exclude sensor D7	6.14	7.21	7.20	7.20
exclude sensor D8	6.15	7.21	7.22	7.20
F-value from ANOVA	0.0222	0.0681	0.2161	0.3635
p-value from ANOVA	1.0000	0.9998	0.9882	0.9400
*Coniferous stands*				
all 8 sensors	5.88	6.17	5.85	5.69
exclude sensor C1	5.84	6.06	5.71	5.61
exclude sensor C2	5.90	6.16	5.85	5.68
exclude sensor C3	5.92	6.15	5.90	5.71
exclude sensor C4	5.81	6.19	5.83	5.66
exclude sensor C5	5.97	6.16	5.89	5.70
exclude sensor C6	5.83	6.12	5.81	5.64
exclude sensor C7	5.80	6.21	5.80	5.68
exclude sensor C8	5.94	6.32	5.98	5.80
F-value from ANOVA	0.0490	0.1967	0.3269	0.2296
p-value from ANOVA	0.9999	0.9914	0.9561	0.9856
We calculated mean temperature using all eight sensors, and compared that with means calculated by excluding one sensor at a time. We tested for significant differences using Analysis of Variance (ANOVA), and those results are reported here.				
